# Involvement of the Right Dorsolateral Prefrontal Cortex in Numerical Rule Induction: A Transcranial Direct Current Stimulation Study

**DOI:** 10.3389/fnhum.2020.566675

**Published:** 2020-12-23

**Authors:** Yuzhao Yao, Xiuqin Jia, Jun Luo, Feiyan Chen, Peipeng Liang

**Affiliations:** ^1^Bio-X Laboratory, Department of Physics, Zhejiang University, Hangzhou, China; ^2^Department of Radiology, Beijing Chaoyang Hospital, Capital Medical University, Beijing, China; ^3^Center for Economic Behavior and Decision-making (CEBD), and School of Economics, Zhejiang University of Finance and Economics, Hangzhou, China; ^4^School of Psychology, Beijing Key Laboratory of Learning and Cognition, Capital Normal University, Beijing, China

**Keywords:** checking effect, cognitive process, right dorsolateral prefrontal cortex, transcranial direct current stimulation, rule induction

## Abstract

Numerical inductive reasoning has been considered as one of the most important higher cognitive functions of the human brain. Importantly, previous behavioral studies have consistently reported that one critical component of numerical inductive reasoning is checking, which often occurs when a discrepant element is discovered, and reprocessing is needed to determine whether the discrepancy is an error of the original series. However, less is known about the neural mechanism underlying the checking process. Given that the checking effect involves cognitive control processes, such as the incongruent resolution, that are linked to the right dorsolateral prefrontal cortex (DLPFC), this study hypothesizes that the right DLPFC may play a specific role in the checking process. To test the hypothesis, this study utilized the transcranial direct current stimulation (tDCS), a non-invasive brain stimulation method that could modulate cortical excitability, and examined whether and how the stimulation of the right DLPFC *via* tDCS could modulate the checking effect during a number-series completion problem task. Ninety healthy participants were allocated to one of the anodal, cathodal, and sham groups. Subjects were required to verify whether number sequences formed rule-based series, and checking effect was assessed by the difference in performance between invalid and valid conditions. It was found that significantly longer response times (RTs) were exhibited in invalid condition compared with valid condition in groups of anodal, cathodal, and sham tDCS. Furthermore, the anodal tDCS significantly shortened the checking effect than those of the cathodal and sham groups, whereas no significantly prolonged checking effect was detected in the cathodal group. The current findings indicated that anodal tDCS affected the process of checking, which suggested that the right DLPFC might play a critical role in the checking process of numerical inductive reasoning by inhibiting incongruent response.

## Introduction

Numerical inductive reasoning, which refers to the ability to identify and extrapolate a general rule or relation from a set of specific numerical elements, has been considered as one of the most important higher cognitive functions of the human brain (Spearman, 1923). This ability has been reported to be closely associated with a wide range of cognitive capabilities, such as school achievement (Díaz-Morales and Escribano, 2013), language processing (Csapó and Nikolov, 2002), and cognitive control (Primi, 2001). Importantly, the number-series completion problem task, for instance, predicting the next number in the sequence, is a typical task that has been widely used in examining numerical inductive reasoning ability. Prior psychological studies have suggested that the processes of solving the number-series completion problems consist of four separate components: detection of relations, discovery of periodicity, description of patterns, and extrapolations (Holzman et al., 1983; Girelli et al., 2004).

The first component, detection of relations, refers to the examination of the number series and the generation of a hypothesis about the number relations among adjacent elements. Relations among numbers may vary considerably in the type of arithmetic operations and the magnitude of the operation. This component is of particular importance because it is an elementary case of rule induction and it is also involved in the operational processes of the second and third components. Importantly, Lefevre and Bisanz (1986) proposed a more detailed model for this relations detection component, which may be useful for explicating the nature of inductive reasoning as well as providing a cognitive basis for detecting individual differences in inductive reasoning ability. According to this model, three sub-procedures—recognition of memorized numerical series, calculation, and checking—are involved in the detection of number relations. Specifically, recognition of memorized numerical series represents a rapid retrieval process of sequence in semantic memory (e.g., “1 2 3 4”), which is referred to as memorized counting series (valid series). In contrast, unfamiliar sequences referred to as non-counting series (valid series), such as “2 5 8 11,” cannot be directly retrieved from semantic memory. In this situation, the second procedure, calculation, is evoked to detect the relations. When a discrepant element appears in the number sequence, such as “1 2 3 5” (invalid series), there are no valid rules that can be concluded to relate all the number elements. The third procedure, checking effect will occur to determine whether the discrepancy is simply an error of the original series (e.g., encoding or calculating mistake; Lefevre and Bisanz, 1986). Generally, checking effect should be larger when the discrepant element differs only slightly from the expected value (e.g., “1 2 3 4”) than when it is completely anomalous (e.g., “1 2 3 79”).

As is found by previous studies, the right dorsolateral prefrontal cortex (DLPFC) plays a fundamental role in top-down regulatory processes of cognitive control (Botvinick et al., 2001; Nunez et al., 2005; Vanderhasselt and Raedt, 2009; Cieslik et al., 2013; Gbadeyan et al., 2016; Boschin et al., 2017), especially solving the conflicting effects in the Stroop (Vanderhasselt et al., 2007; Boschin et al., 2017; Masina et al., 2018; Seok and Sohn, 2018) and Flanker tasks (Gbadeyan et al., 2016; Chen et al., 2018). While the checking process reflects the conflict cost (Li et al., 2009) on performance of relations detection, it is assessed as the difference in response time (RT) between invalid and valid trials. Our eye movement study has revealed significantly longer fixation duration in the invalid task, which indicates that checking effect of cognitive control suppresses the original response (Fu et al., 2014). Taken together, we hypothesize that the right DLPFC may play a critical role in the checking process of the relations identification.

Transcranial direct current stimulation (tDCS), a non-invasive, low current brain stimulation technique, can allow for causal inferences between the stimulated brain region and the corresponding behavioral performance. Brain function can be temporarily and reversibly modulated by active stimulation (Nitsche and Paulus, 2001). The aim of the present study was to examine the involvement of the right DLPFC in the checking process. It was particularly postulated that compared with the sham tDCS (StDCS), anodal tDCS (AtDCS) over the right DLPFC would affect cognitive control of checking by decreasing response latency, whereas cathodal tDCS (CtDCS) would affect checking by increasing response latency. Three kinds of tasks were designed including valid, invalid, and anomalous series. The valid series consisted of four elements that could be described by a simple rule (e.g., “1 2 3 4”). The fourth element had a slight difference that violated the simple rule from valid series in invalid series (e.g., “1 2 3 5”) and a large difference in anomalous series (e.g., “1 2 3 79”). On the basis, the anomalous series was designed to eliminate the potential possibility that the involvement of the right DLPFC was just due to “irregularity” of discrepant sequences. To simplify and purify the question, only counting series tasks (e.g., “1 2 3 4” vs. “1 2 3 5” vs. “1 2 3 79” for valid, invalid, and anomalous series) were employed.

## Materials and Methods

### Ethics Statement

This study was approved by the Institutional Review Board of Zhejiang University and conducted in compliance with the Declaration of Helsinki. All subjects provided written informed consents before the whole experiments.

### Participants

We recruited a total of 90 college students (mean age = 22.64 ± 1.95 years, 54 males) from Zhejiang University. The participants were required to be: (1) healthy; (2) right-handed; (3) with normal or corrected-to-normal vision; (4) without metal in the head or pacemaker; (5) free of any medication at the time of the experiment; (6) without personal or family history of epilepsy; and (7) without brain injury or history of neurological/psychiatric disorders. They were informed about the potential itching sensation of tDCS and were then randomized to receive either anodal, cathodal, or sham tDCS on the right DLPFC. A total of nine subjects (three males) were not included in the final analyses for fatigue caused by inadequate sleep or incomplete behavioral assessments. Hence, the anodal, cathodal, and sham groups in the final sample for data statistics had 27 (mean age = 22.81 ± 1.71 years, education year = 16.15 ± 1.35 years, 18 males), 27 (mean age = 22.56 ± 1.83 years, education year = 16.19 ± 1.18 years, 18 males), and 27 (mean age = 22.56 ± 2.33 years; education year = 16.11 ± 1.72 years, 15 males) participants, respectively (see Table 1). A one-way ANOVA showed that the three groups did not differ significantly in age (*F*_(2,78)_ = 0.16, *p* = 0.86) or education (*F*_(2,78)_ = 0.02, *p* = 0.98). *χ*^2^ tests showed that the three groups were matched in gender distribution (*χ*^2^ = 0.95, *p* = 0.62). All participants were paid 40 CNY for participating in the experiment.

**Table 1:** The overall mean and standard deviations of the demographic information.

	AtDCS	CtDCS	StDCS
Subject	27	27	27
Age	22.81 (1.71)	22.56 (1.83)	22.56 (2.33)
Education year	16.15 (1.35)	16.19 (1.18)	16.11 (1.72)

### tDCS

**Figure 1:**
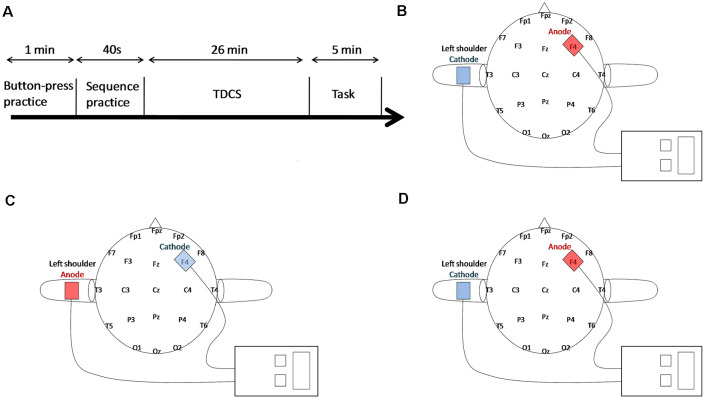
Experimental paradigm. **(A)** The experimental procedure design. **(B)** For Anodal tDCS (AtDCS), the anode was over the right Dorsolateral Prefrontal Cortex (DLPFC), whereas the cathode was over the left shoulder. **(C)** For Cathodal tDCS (CtDCS), the electrodes were reversed. **(D)** For Sham tDCS (StDCS), the placement of electrodes was the same as AtDCS.

The tDCS was delivered by a battery-driven DC-Stimulator Plus (neuroConn, Germany) through a pair of 5 × 7 cm saline-soaked sponge electrodes. In all three groups, the active electrode was placed over the right DLPFC (F4, based on the 10-20 international electroencephalogram system for electrode placement), whereas the reference electrode was placed on the left shoulder to avoid extra stimulation in the brain cortex (see Figure 1; Berryhill et al., 2010). For the anodal and sham groups, the anode that was placed over F4 was the active electrode, whereas for the cathodal group, the electrodes were reversed, and the cathode was placed over F4. The current strength was 1.5 mA (current density of 0.043 mA/cm^2^) lasting for 25 min in both the active groups with a ramping up and down period of 30 s at the beginning and at the end of the stimulation, respectively. In the sham group, the current lasted only for 30 s with both the same strength of 1.5 mA and the period of ramping up and down. The participants were blind to the tDCS condition to better observe the stimulation effect and to deal with the unexpected situation of the stimulation. During tDCS, all participants were comfortably seated and required to keep relaxed with their eyes closed. The experimental task began immediately after stimulation.

### Experimental Task

The paradigm was adapted from a previous study by Lefevre and Bisanz (1986). Three kinds of counting series tasks, valid, invalid, and anomalous, were designed, and examples were shown in Table 2. The valid series consisted of four elements and could be described by a simple rule and retrieved rapidly and directly from semantic memory, such as “1 2 3 4.” Each valid counting series listed in Table 2 was presented four times, for a total of 24 trials. Both invalid and anomalous series were derivatives of valid counting series. For the invalid series, the fourth element of the sequence was slightly different from the correct counterpart (i.e., +1 to the correct counterpart) in which the checking process would be involved, such as “1 2 3 5,” whereas for the anomalous series, the fourth element had a very large number, such as “1 2 3 79.” The order of the series was randomized with the constraint that no more than three valid series or three invalid series appeared consecutively. Accordingly, checking effect was assessed by the difference in RT between invalid and valid conditions (i.e., checking = RT_invalid_ − RT_valid_; Lefevre and Bisanz, 1986).

**Table 2:** Examples of experimental tasks.

	Valid	Invalid	Anomalous
Sequences	1 2 3 4	1 2 3 5	1 2 3 84
	1 3 5 7	1 3 5 8	1 3 5 86
	2 3 4 5	2 3 4 6	2 3 4 78
	2 4 6 8	2 4 6 9	2 4 6 84
	3 4 5 6	3 4 5 7	3 4 5 92
	4 5 6 7	4 5 6 8	4 5 6 73

Subjects were required to verify whether the number series formed a simple rule-based sequence (i.e., valid or invalid) by two response buttons. The left and right index fingers were assigned to response keys “F” and “J” in the keyboard, respectively. Subjects were randomly assigned and counterbalanced to the left-valid group (i.e., “F” represented valid) or the right-valid group (i.e., “J” represented valid). Before the stimulation, a button-press practice task with 24 trials (12 valid trials and 12 invalid trials) was conducted to measure general response speed for each participant, and a number-series practice task with nine trials (three valid trials, three invalid trials, and three anomalous trials) was carried out to get subjects familiar with the experiment. Previous study had demonstrated that individual differences exhibited in processing speed even in young individuals (Magistro et al., 2015). Accordingly, to eliminate the effect of the different response speeds among groups on the final findings, additional analysis on button-press practice block was further performed. In the button-press task, the Chinese word “
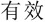
” or “
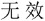
” would be presented in the screen that represented valid or invalid, respectively. Participants were required to respond to the condition as quickly and accurately as possible with their keyboards. Accuracy and RT of the correct response trials were recorded for measuring the group difference about response speed.

**Figure 2:**
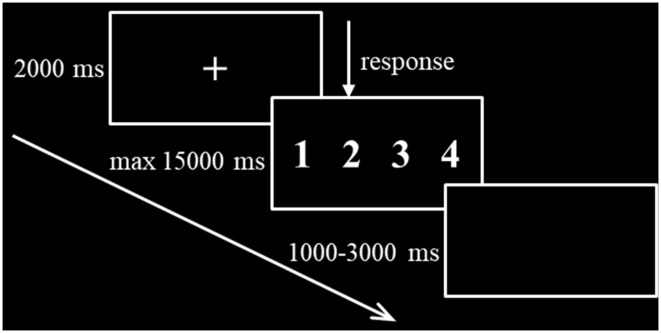
Stimulus presentation paradigm.

The formal task session began immediately after the stimulation. As shown in Figure 2, the task presentation paradigm was as follows: first, a central fixation cross was presented for 2,000 ms in the black background, followed by a number series that was presented for a maximum of 15,000 ms with a randomized interstimulus interval between 1,000 and 3,000 ms. The subject was required to respond to the sequence as quickly and accurately as possible. After the button press, the sequence was replaced immediately by a central fixation cross. The experiment lasted for approximately 5 min and consisted of 24 valid trials, 12 invalid trials, and 12 anomalous trials that were presented randomly by the E-Prime 2.0 software. Accuracy and RT of the correct response trials were recorded and analyzed.

### Statistical Analysis

All behavioral analyses were conducted using SPSS 22.0. A 3 (Condition: valid, invalid, anomalous) × 3 (Group: anodal, cathodal, sham) repeated measures ANOVA was first carried out to test whether there were significant Condition or Group differences in RT or accuracy during the number-series task. If sphericity assumptions were violated, Greenhouse–Geisser adjustments of the *p* values were reported. Our main interest concerned the checking process during the number-series task. It was assessed by the difference in response time RT between invalid and valid conditions (i.e., checking = RT_invalid_ − RT_valid_; Lefevre and Bisanz, 1986). The smaller the checking effect index, the better the checking efficiency. Then, one-way ANOVA with the “group” (i.e., AtDCS, CtDCS, and StDCS) as between-group variable was performed on this checking index to test whether there were significant group differences. *Post-hoc* *t*-tests were conducted using the Bonferroni correction for multiple comparisons.

## Results

Analysis of the response speed through button-press practice block showed that no significant differences were revealed in the button-press speed among the three groups (*F*_(2,78)_ = 0.78, *p* = 0.46). Along with the comparable demographic information, as a result, general differences that might have an effect on the formal experiment did not exist, and the three groups were comparable to some extent.

**Figure 3:**
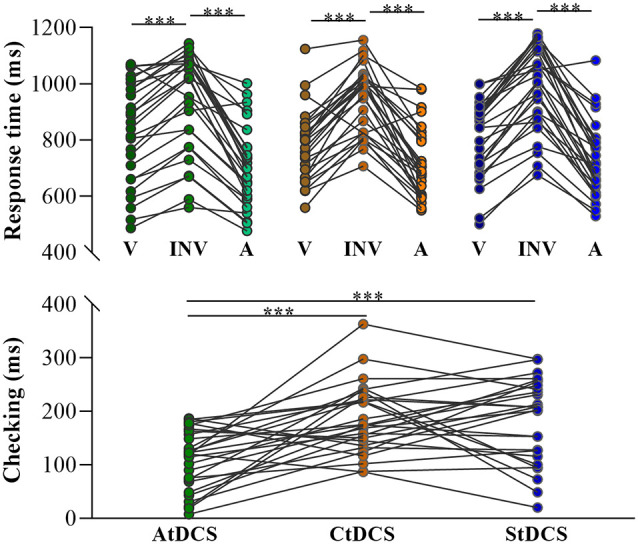
Response time (RT) for the three conditions and checking effect (i.e., invalid–valid) of the three transcranial direct current stimulation (tDCS) groups. V, valid; INV, invalid; A, anomalous; green scatters, AtDCS; yellow scatters, CtDCS; blue scatters, StDCS; ***p < 0.001.

The overall mean and standard deviations of the results were shown in Table 3. Mean RT was not significantly correlated with accuracy in any condition or any group (smallest *p* = 0.17), indicating no speed–accuracy trade-off in the formal number-series task (see Table 4). The 3 (Condition: valid, invalid, anomalous) × 3 (Group: anodal, cathodal, sham) repeated measures ANOVA on accuracy revealed a significant main effect of Condition (*F*_(2,156)_ = 40.43, *p* < 0.001, partial *η*^2^ = 0.34). *Post-hoc* tests with Bonferroni correction showed that accuracy in the anomalous condition was significantly higher than accuracy in the valid (*p* = 0.002) and invalid conditions (*p* < 0.001). Additionally, accuracy in the valid condition was significantly higher than accuracy in the invalid condition (*p* < 0.001). There was no significant main effect of Group (*F*_(2,78)_ = 0.09, *p* = 0.92, partial *η*^2^ = 0.002) or Condition-by-Group interaction (*F*_(4, 156)_ = 0.08, *p* = 0.99, partial *η*^2^ = 0.002). A parallel repeated measures ANOVA on RT also revealed a significant main effect of Condition (*F*_(2,156)_ = 221.82, *p* < 0.001, partial *η*^2^ = 0.74). *Post-hoc* tests with Bonferroni correction showed that RT in the anomalous condition was significantly shorter than RT in the valid (*p* < 0.001) and invalid conditions (*p* < 0.001). Moreover, RT in the valid condition was significantly shorter than RT in the invalid condition (*p* < 0.001, Figure 3), suggesting the presence of checking effect on RT. There was no significant main effect of Group (*F*_(2,78)_ = 0.16, *p* = 0.85, partial *η*^2^ = 0.004). Interestingly, there was a significant Condition-by-Group interaction (*F*_(4,156)_ = 2.56, *p* = 0.04, partial *η*^2^ = 0.06). When participants’ age, gender, and education levels were included in the above ANOVA models as covariates, the results remained similar.

**Table 3:** The overall mean and standard deviations of the results.

	AtDCS	CtDCS	StDCS
Accuracy			
Valid	0.978 (0.033)	0.980 (0.031)	0.981 (0.027)
Invalid	0.920 (0.085)	0.926 (0.078)	0.917 (0.098)
Anomalous	0.991 (0.027)	0.994 (0.022)	0.994 (0.022)
Response time (ms)			
Valid	836 (184)	781 (125)	805 (136)
Invalid	946 (187)	963 (127)	988 (150)
Anomalous	702 (150)	719 (128)	730 (134)

*Note: data were expressed as mean (STD). AtDCS, anodal tDCS; CtDCS, cathodal tDCS; StDCS, sham tDCS*.

**Table 4:** Correlations between accuracy and response time (RT) in each task condition and each group.

	Anodal	Cathodal	Sham
Valid	0.11	0.27	0.27
Invalid	0.21	0.19	0.26
Anomalous	0.03	−0.07	−0.13
Checking effect	−0.22	−0.01	0.11

To further examine whether this interaction effect was driven by a group difference in checking effect, a one-way ANOVA with Group (anodal, cathodal, sham) as between-group variable was applied on the checking index. Interestingly, there was a significant main effect of Group (*F*_(2,78)_ = 9.86, *p* < 0.001, partial *η*^2^ = 0.20). *Post-hoc* tests with Bonferroni correction showed that the checking effect index on RT in the anodal group was significantly smaller than those in both the cathodal (*p* < 0.001) and sham groups (*p* < 0.001), indicating that the AtDCS reduced the differences in performance between valid and invalid conditions. Therefore, AtDCS might affect the process of checking by reducing the RT differences between invalid and valid conditions (Figure 3). Considering the significant Condition-by-Group interaction in RT, a further independent-samples *t*-test was conducted in the valid and invalid conditions between the anodal and sham groups to figure out whether the differences were derived from prolonged RT of the valid condition or reduced RT of the invalid condition. However, no significant differences were found in valid (*t*_(52)_ = 0.70, *p* = 0.49) or invalid conditions (*t*_(52)_ = 0.91, *p* = 0.37). The checking effect index on RT showed no significant group difference (*p* = 0.99) between the cathodal and sham groups. When participants’ age, gender, and education levels were included in the above analyses as covariates, the results remained similar.

## Discussion

The present study aimed to provide a fundamental understanding of neural mechanisms for a more detailed account of the cognitive processes involved in solving the number-series task by means of tDCS. Particularly, it was found that the AtDCS over the right DLPFC affected the performance of the checking process during relations detection, whereas the CtDCS over the right DLPFC did not significantly affect the checking performance to some extent. The current findings indicate that the right DLPFC is involved in the checking process of relations detection during simple inductive reasoning, which further suggest that the dual-process is involved in numerical inductive reasoning by the deliberate and effortful System 2 inhibiting the rapid and automatic System 1 processes (Evans, 2003; Liang et al., 2014a).

It was noticed that in contrast to the excitation effects with anodal neuromodulation, the cathodal stimulation did not achieve a significant inhibition effect on checking in the present study. The heterogeneity of cathodal stimulation may account for this result. In fact, the lack of inhibitory cathodal effect has been reported previously, probably due to the compensation processes during cognitive task (Jacobson et al., 2012). In particular, a meta-analysis has pointed out that no inhibitory effect exhibited in the cathodal tDCS over the DLPFC compared with sham tDCS (Dedoncker et al., 2016), which may account for the finding in the current study. The cathodal-inhibition effect on the checking process may be explicitly explored by transcranial magnetic stimulation (TMS) in further studies.

Alternatively, the involvement of the right DLPFC in the checking process may be due to the rule complexity rather than the incongruent inhibition. Our previous study has demonstrated that more neural resources in the right DLPFC would be recruited to support the integration of multiple relations when the rule complexity of number relations increases (Jia et al., 2015). However, when the behavior performance was included as nuisance variate of no interest, the effect of anodal tDCS on the checking process still remained (*p* < 0.001), which further ruled out the potential effect of rule complexity in the current findings.

The current finding could not neglect the role of the left DLPFC in the more detailed account of the cognitive processes involved in number-series tasks of relations detection. Previously, neuroimaging studies have demonstrated that the left DLPFC, together with the posterior parietal cortex (PPC), provides a primary support for the declarative knowledge retrieval, integration, and imaginary representation (Jia et al., 2011, 2015; Liang et al., 2014b, 2016). In fact, the recruitment of the bilateral DLPFC is distinct and collaborative with each other to specific cognitive function. In the present study, during the checking process, participants would be aware of the error (i.e., slight discrepancy), and a re-process was engaged under cognitive control of the right DLPFC (Nunez et al., 2005; Vanderhasselt et al., 2007; Vanderhasselt and Raedt, 2009; Cieslik et al., 2013; Gbadeyan et al., 2016; Boschin et al., 2017). Moreover, our previous patient study has revealed that patient with mild cognitive impairment (MCI) exhibited an increased collaboration between the left and the right DLPFC for identification of relations to compensate for the neurodegenerative course of this disease (Yang et al., 2009). As the conflict cost, the checking effect would be present in the invalid condition rather than the valid condition. However, it was a limitation of a between-subject design to clarify the direction of the observed tDCS effect since no significant difference was found in both valid and invalid conditions between the anodal and sham groups. A further study of within-subject design would be addressed to verify this issue. Therefore, it was found that with AtDCS over the right DLPFC, reduced differences in RT between the valid and invalid conditions might reflect better cognitive control, which provided the evidence for a critical role of the right DLPFC in checking of relations detection.

It should be mentioned that, as a non-invasive neuromodulation technique, tDCS can investigate the contribution of brain regions to specific cognitive functions (Schulz et al., 2013). Brain function can be temporarily and reversibly modulated by active stimulation (Nitsche and Paulus, 2001), such as the enhancement of cognitive functions in humans (Fregni et al., 2005). Previous studies had focused on the amelioration of cognitive control by tDCS over DLPFC (Feeser et al., 2014), especially in patients with major depressive disorder (Wolkenstein and Plewnia, 2013; Brunoni et al., 2014). The current finding provides further evidence that the inductive reasoning ability could be strengthened through the neuromodulation of tDCS.

The current study has several limitations. First, due to methodological issues of tDCS, cathodal-inhibition effect in tDCS may be not significant. Thus, further studies are required to establish the causal relationship between the right DLPFC and its function by TMS. Second, the present study utilized a cross-sectional between-group design, which cannot exclude the influence of individual differences. However, the current finding is less affected as the checking effect representing a relative index. Future research should consider better experimental designs to address this issue more rigorously. In addition, previous tDCS studies have demonstrated that tDCS is state-dependent by tasks (Bikson and Rahman, 2013; Schroeder and Plewnia, 2017) and easy tasks may interact with tDCS-induced electric fields differentially compared with difficult ones. To understand in-depth the neural mechanisms of numerical inductive reasoning, more difficult tasks would help to speculate whether the present tDCS results would generalize to other test situations with more cognitive resources involved. Finally, in the recruitment, we did not put anxiety and depression as the exclusion criteria so that there could be a slight limitation to our experiment.

Taken together, the current findings demonstrate the important role of the right DLPFC in the checking process of relations detection, which would further subserve to establish its computational neurocognitive model under a more detailed account of the cognitive processes involved in solving the rule induction tasks.

## Data Availability Statement

The raw data supporting the conclusions of this article will be made available by the authors, without undue reservation.

## Ethics Statement

The studies involving human participants were reviewed and approved by The Institutional Review Board of Zhejiang University. The patients/participants provided their written informed consent to participate in this study.

## Author Contributions

PL, FC, XJ, and YY contributed to the study conception and design. Data were collected by YY and analyzed and interpreted by YY and XJ. The drafting of the manuscript was written by YY and XJ and was critically revised by PL, FC, and JL. At last, the final approval of the version to be published was confirmed by all the authors. All authors contributed to the article and approved the submitted version.

## Conflict of Interest

The authors declare that the research was conducted in the absence of any commercial or financial relationships that could be construed as a potential conflict of interest.
